# Uric Acid to Platelet Ratio (UAPR) and Its Link to Lipid Abnormalities: Findings from a Large Cohort Study in Saudi Arabia

**DOI:** 10.3390/jcm14175952

**Published:** 2025-08-22

**Authors:** Yazeed Alshuweishi, Lama Izziddeen, Muath Alsaidan, Noha A. Alshuwayer, Faisal A. Alshuweishi, Ahmed M. Basudan

**Affiliations:** 1 Department of Clinical Laboratory Sciences, College of Applied Medical Sciences, King Saud University, Riyadh 12372, Saudi Arabia; 445920965@student.ksu.edu.sa (L.I.); ahmbasudan@ksu.edu.sa (A.M.B.); 2 Department of Family and Community Medicine, College of Medicine, King Saud University, Riyadh 12372, Saudi Arabia; malsaidan@ksu.edu.sa; 3 Department of Anatomy, College of Medicine, King Saud University, Riyadh 11461, Saudi Arabia; nohamd@ksu.edu.sa; 4 Department of Pathology and Laboratory Medicine, King Khalid University Hospital, King Saud University Medical City, Riyadh 12372, Saudi Arabia; falshuweishi@ksu.edu.sa

**Keywords:** UAPR, dyslipidemia, cardiovascular disease, Saudi Arabia

## Abstract

**Background:** Lipid disturbance is a hallmark of cardiometabolic abnormalities and a primary contributor to cardiovascular disease risk. Immunometabolic markers show promise for better risk classification. This study evaluated the uric acid to Platelet ratio (UAPR) as lipid abnormality marker in a broad cohort of Saudi adults. **Methods:** Data from 7781 adults in the Elta Medical Laboratory database were analysed. Subjects were stratified by lipid status, and UAPR levels were analyzed. Additionally, lipid abnormality distribution across UAPR tertiles and risk profiles, including ROC analysis, were evaluated. **Results:** UAPR were markedly increased in patients with abnormal lipid profiles while high UAPR (H-UAPR) subjects showed multiple dyslipidemic patterns including elevated levels of triglycerides (TG), low-density lipoprotein (LDL-C), non-high-density lipoprotein (non-HDL-C), and remnant cholesterol (RC), alongside reduced HDL-C levels. Notably, UAPR correlated with all lipid parameters, most strongly and inversely with HDL-C (r = −0.314) and remained independently associated with TG and HDL-C in multivariable regression. Consistently, H-UAPR was common across all dyslipidemic forms, especially low HDL-C, nearly twice as frequent as in N-UAPR (52.4% vs. 35.0%). The odds were specifically increased for low HDL-C (OR = 2.02, *p* < 0.0001) and a high TC/HDL-C ratio (OR = 2.94, *p* < 0.0001) in H-UAPR patients. ROC analysis demonstrated that UAPR had moderate yet significant diagnostic performance, particularly for identifying high TC/HDL-C (AUC = 0.671, *p* < 0.001) and HDL-C (AUC = 0.618, *p* < 0.001). **Conclusions:** UAPR shows considerable promise as an immunometabolic marker linked to various dyslipidemic forms with potential for hyperlipidemia screening and stratification, warranting further validation.

## 1. Introduction

Disrupted lipid homeostasis is a recognized and modifiable contributor to atherosclerosis and cardiovascular disease, posing a major global health burden [1]. It is characterized by elevated triglycerides (TG) and low-density lipoprotein cholesterol (LDL-C), alongside reduced high-density lipoprotein cholesterol (HDL-C) [2]. Key drivers of acquired dyslipidemia include sedentary lifestyle and obesity, as excess adiposity, insulin resistance, and chronic inflammation promote the release of free fatty acids (FFAs) into the circulation, leading to lipotoxicity in the liver, skeletal muscle, and myocardium [3,4,5]. This heightened FFA flux to the liver promotes hypertriglyceridemia, excessive very low-density lipoprotein (VLDL) production, increased LDL-C synthesis, and reduced HDL-C, changes further aggravated by proinflammatory cytokines secreted by VAT [6,7]. Ultimately, these alterations drive the development of secondary dyslipidemia, which may begin as early as young adulthood [8,9,10], highlighting the importance of understanding its determinants and underlying pathophysiological mechanisms to inform effective prevention and management strategies.

Globally, the prevalence of dyslipidemia among adults ranges from 20% to 80%, influenced by geography, dietary patterns, and socioeconomic status [11]. In Saudi Arabia, prevalence ranges from 20–40% nationally, reaching over 60% in some regions such as Jeddah, with the burden being especially high among individuals with type 2 diabetes, where up to two-thirds are affected [12,13]. Dyslipidemia often clusters with obesity, hypertension, and impaired glucose metabolism, reflecting shared mechanisms of insulin resistance, visceral adiposity, and lifestyle-related risk factors, while men, older adults, those with lower education, and residents of certain regions are disproportionately affected [12]. Despite this considerable burden, a large proportion of at-risk individuals remain undiagnosed, untreated, or inadequately controlled, with reports indicating that only 10.5–47.3% of dyslipidemic patients were aware of their condition, 17.8% formally diagnosed, 40.0–94.0% treated, 45.0–77.4% adherent, and just 28.0–41.5% achieving control [14]. Such figures highlight the gap between the high prevalence of dyslipidemia and limited detection, underscoring the need for early identification and risk stratification to reduce future complications.

Dyslipidemia is increasingly recognized not only as a disorder of lipid imbalance but also as an inflammatory and metabolic disease, underpinned by mechanisms such as oxidative stress, adipose tissue dysfunction, and immune activation [15]. In this context, uric acid and platelets both contribute to the inflammatory state and thrombotic event via secreting inflammatory and thrombotic mediators [16,17,18,19]. Uric acid, a purine metabolism byproduct, is mainly excreted by the kidneys and imbalance due to overproduction or reduced excretion lead to a number of diseases including gout, kidney stones and urate nephropathy [20,21,22,23]. However, more recent studies demonstrated that hyperuricemia is not only exclusively a consequence of impaired renal function, but it is strongly linked with cardiometabolic conditions such as raised blood pressure, dysglycemia and metabolic syndrome [24,25,26]. It was shown that increased uric acid levels is closely associated with the accumulation of VAT [27] and is implicated in promoting insulin resistance and persistent low-grade inflammation, further amplifying cardiovascular risk [28,29,30]. Additionally, platelet number and activity reflect not only thrombotic potential but also the underlying inflammatory milieu [31]. Platelets can modulate immune responses, release pro-inflammatory cytokines, and influence lipid metabolism through interactions with endothelial cells and macrophages [18,32]. Platelet-related indices were significantly altered in metabolic conditions such as hyperglycemia and dyslipidemia highlighting their utility as metabolic and cardiovascular risk markers [33,34,35,36]. Taken together, the interplay between elevated uric acid and altered platelet dynamics may serve as a critical link between metabolic dysregulation inflammation and cardiovascular risk.

The high prevalence of dyslipidemia in Saudi Arabia, together with its asymptomatic nature, highlights the importance of identifying blood-derived indices linked to this condition to enhance primary and secondary prevention strategies. In recent years, elevated levels of inflammatory biomarkers and blood-derived indices such as interleukin 6, ceramides, serum cystatin C, hemoglobin albumin lymphocyte platelet (HALP) score, platelet lymphocyte ratio (PLR) and platelet monocyte ration (PMR) have been associated with dyslipidemia and metabolic disturbance [37,38,39,40,41]. These indices have proved useful in identifying individuals at higher risk of cardiometabolic complications However, evidence regarding the potential role of the uric acid-to-platelet ratio (UAPR) in this context is currently lacking. This study aims to establish this association and to characterize the patterns of UAPR across different dyslipidemia phenotypes in a large Saudi population. In addition, the study seeks to evaluate the modifying effects of sex and age on the association estimates, as well as to assess the diagnostic accuracy of UAPR for a comprehensive range of lipid parameters and related ratios.

## 2. Methods

### 2.1. Study Design

A retrospective assessment was carried out using laboratory data collected from Elta Medical Laboratories between January 2023 and December 2024. The objective is to examine the linkage between the uric acid to platelet ratio (UAPR) and various lipid parameters and atherogenic lipid ratios. The study protocol and design were reviewed by the Institutional Review Board at King Saud University, Riyadh, Saudi Arabia (Approval No: E-25-9893). As the study used de-identified routine laboratory data, informed consent was waived.

### 2.2. Data Collection

A total of 8234 subjects were initially extracted from the Elta Medical Laboratory database. After applying the exclusion criteria, primarily the absence of complete lipid profiles or missing uric acid and platelet count data, 7781 subjects were retained for the final analysis. The collected dataset included demographic information such as age and sex, hematological parameters and biochemical data comprising serum uric acid, a full lipid panel. This includes measurements of triglycerides (TG), total cholesterol (TC) and high-density lipoproteins. Low-density lipoprotein was calculated using the Friedewald formula. Non-HDL-C was derived by subtracting HDL-C from TC, while remnant cholesterol (RC) was estimated as the difference between TC, HDL-C, and LDL-C. The Lipoprotein Combine Index (LCI) was calculated using the formula: LCI = (Triglycerides mmol/L × Total Cholesterol mmol/L × LDL-C mmol/L)/HDL-C mmol/L, as previously described [42]. Lipid abnormalities were defined based on deviations from established reference ranges for individual lipid parameters including: TC ≥ 200 mg/dL, LDL-C ≥ 130 mg/dL, HDL-C < 40 mg/dL in males and <50 in females, non-HDL-C ≥ 150, RC ≥ 24, TG ≥ 150 mg/dL, LCI ≥ 16 [42,43,44,45,46]. Lipid-related ratios were considered elevated based on the following thresholds: TC/HDL-C ratio ≥ 5, LDL-C/HDL-C or TG/HDL-C ratios ≥ 3, and RC/HDL-C ratio ≥ 0.56 [42,43,44,45,46]. UAPR was calculated according to the following formula: (uric acid (mg/dL) ÷ platelet count (×10^9^/L)). Due to the lack of standardized clinical cut-off values for UAPR, values were divided into tertiles to facilitate group comparisons while ensuring adequate statistical power across subgroups. The tertile thresholds were as follows: T1 (<1.567), T2 (1.568–2.322), and T3 (>2.322).

### 2.3. Statistical Analysis

All statistical procedures were carried out using GraphPad Prism version 9.0 (GraphPad Software, San Diego, CA, USA). Normality of continuous data was evaluated via Shapiro–Wilk test. Due to non-normal distributions and unequal variances, results were reported as medians with interquartile ranges (IQR), and group comparisons across more than two UAPR tertile groups were conducted using the Kruskal–Wallis test. For two-group comparisons, the Kolmogorov–Smirnov test was used, as it is more sensitive to differences in both central tendency and distribution shape, particularly in large samples. Spearman correlation analysis was used to assess the association between UAPR and individual lipid parameters as well as lipid ratios. To control for multiple testing, *p* values from correlation analyses were adjusted using the Benjamini–Hochberg false discovery rate (FDR) procedure, with significance set at q < 0.05. Multilinear regression analyses were performed GraphPad Prism v10.5.0 (GraphPad Software, CA, USA) to evaluate the associations between variables. Differences in prevalence between males and females were assessed using the Chi-square test of independence. Risk and diagnostic accuracy were assessed by calculating odds ratios (OR), while the ability of UAPR to discriminate individuals with lipid abnormalities was evaluated using receiver operating characteristic (ROC) curve analysis and the corresponding area under the curve (AUC). AUC interpretation was interpreted as follows: <0.6 = poor, 0.6 to 0.7 = moderate, 0.7 to 0.8 = good, and >0.8 = excellent. All statistical tests considered *p* values below 0.05 as significant.

## 3. Results

### 3.1. Clinical and Biochemical Features of the Included Subjects

**Figure 1:**
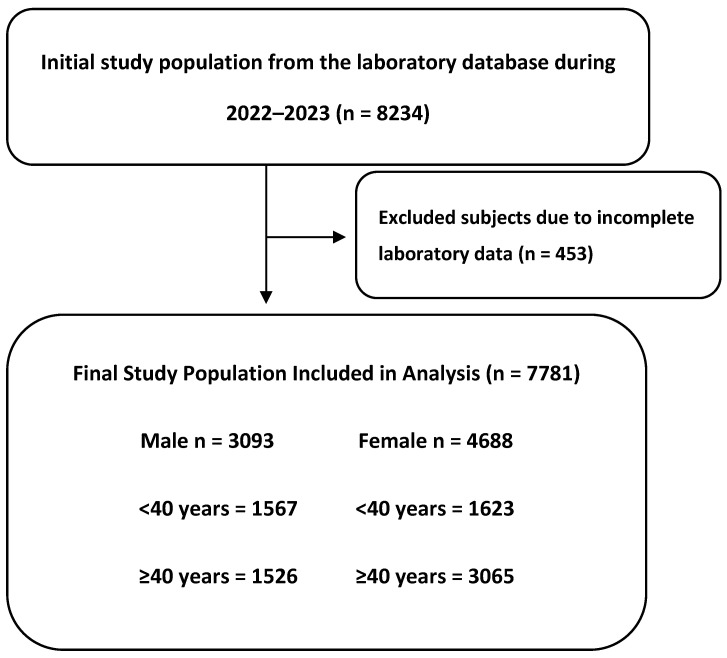
Flow diagram of study population selection.

Of the 8234 records initially identified, 7781 participants were included after excluding those with incomplete laboratory data (Figure 1). The cohort had a median age of 37 years, with females accounting for 60.25% of the study population (Table 1). Key hematological parameters included a hemoglobin level of 14.2 g/dL and a red cell count of 5.24 × 10^12^/μL. Median fasting glucose was 92 mg/dL, while lipid levels were generally within reference ranges. Renal function markers were within normal ranges, with serum creatinine at 0.70 mg/dL (0.60–0.85) and urea at 22 mg/dL (16–27). Liver function markers were normal, with ALT and AST both at 18 U/L.

### 3.2. Prevalence of Lipid Abnormalities and Its Derived Ratios in Males and Females

Among the total study population, the overall prevalence of dyslipidemia varied across lipid parameters and showed notable sex-specific differences (Table 2). The prevalence of low HDL-C showed the most pronounced sex-specific difference among all lipid abnormalities. Overall, 42.2% of the study population had low HDL-C. However, when stratified by sex, only 23.6% of males were affected compared to 54.5% of females. The prevalence of LDL-C abnormality was also high, affecting 37.55% of participants (39.73% males, 36.11% females), followed closely by high TC (35.50%) and high non-HDL-C (36.70%). Elevated TG were present in 14.59% of individuals, again more common in males (15.71%) than females (13.84%). For lipid ratios, 30.90% of the population had a high LDL-C/HDL-C ratio, while 23.99% had an elevated TG/HDL-C ratio. RC was elevated in 23.34%, and high RC/HDL-C ratio was detected in 22.30%. Additionally, 36.49% of subjects had an elevated LCI, with prevalence higher in males (39.80%) than females (34.30%).

### 3.3. Higher UAPR Levels Exhibited Adverse Changes in Lipid Profile and Ratios

Across the tertiles of UAPR, a progressive and significant rise in lipid abnormalities was noted (all *p* < 0.0001). As illustrated in Table 3, TG levels rose progressively from 82 mg/dL in T1 to 106 mg/dL in T3. Similarly, TC increased from 204 mg/dL to 214.3 mg/dL, while LDL-C rose from 97 mg/dL to 102 mg/dL across tertiles. Conversely, HDL-C showed a declining trend, falling from 45 mg/dL in T1 to 37 mg/dL in T3. Moreover, lipid fractions such as non-HDL-C and RC also showed significant upward trends, increasing from 129 mg/dL and 14 mg/dL in the lowest tertile to 146 mg/dL and 18 mg/dL in the highest tertile, respectively.

In parallel, all calculated lipid ratios demonstrated a worsening trend with increasing UAPR. The TG/HDL-C ratio increased from 1.60 to 2.42, LDL-C/HDL-C from 2.19 to 2.86, and TC/HDL-C from 3.49 to 4.31 across tertiles. Similarly, RC/HDL-C rose from 0.26 to 0.41, whereas LCI escalated markedly from 9.65 in T1 to 16.96 in T3.

### 3.4. Subjects with Aberrant Lipid Parameters and Ratios Exhibit a Significant Increase in UAPR

**Figure 2:**
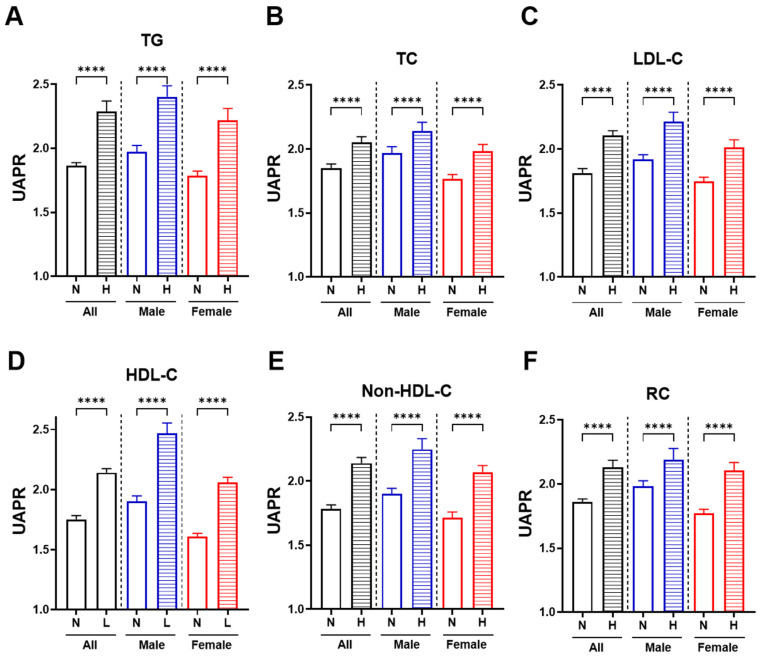
UAPR levels in relation to different lipid abnormalities, stratified by sex. Bar graphs show median UAPR values with interquartile range (±IQR) among individuals with normal (N) and high (H) levels of each lipid parameter across the total population (black bars), males (blue bars), and females (red bars). Panels represent: (**A**) triglycerides (TG), (**B**) total cholesterol (TC), (**C**) LDL-C, (**D**) HDL-C, (**E**) non-HDL-C, and (**F**) remnant cholesterol (RC). Asterisks indicate statistical significance between N and H groups within each sex category (**** *p* < 0.0001).

Figure 2 depicts the sex-stratified relationship between UAPR and various lipid abnormalities. Across all panels (A–F), individuals with dyslipidemia exhibited significantly higher UAPR values compared to those with normolipidemic (*p* < 0.0001). Specifically, UAPR was markedly elevated in individuals with high triglycerides (Figure 2A), total cholesterol (Figure 2B), LDL-C (Figure 2C), non-HDL-C (Figure 2E), and remnant cholesterol (Figure 2F), while it was significantly increased with low HDL-C (Figure 2D), indicating an inverse relationship. This trend was consistent in both males and females, with the most pronounced elevations observed in females with abnormal lipid profiles.

**Figure 3:**
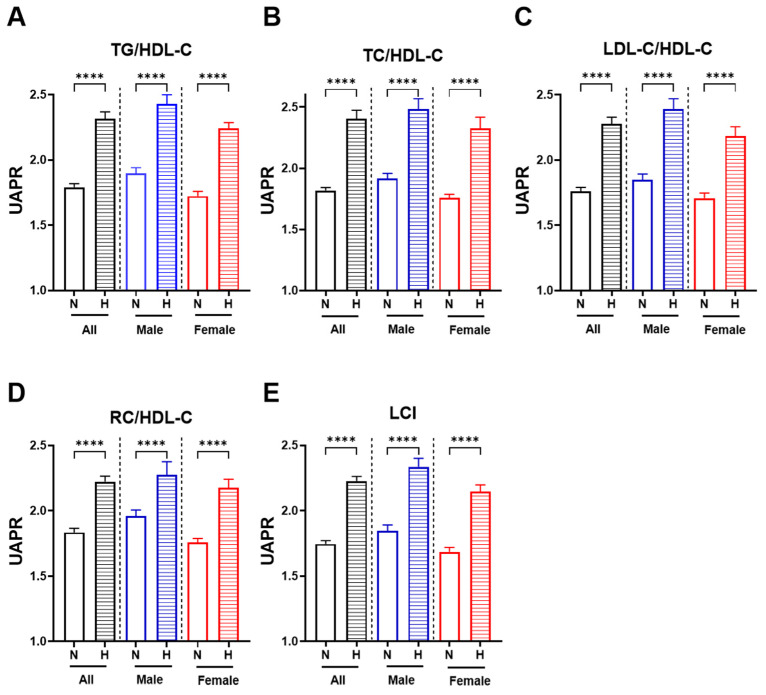
UAPR values in relation to atherogenic lipid ratios, stratified by sex. Bar graphs display the median UAPR values ± interquartile range (IQR) among individuals with normal (N) and high (H) values for each lipid ratio across the total population (black bars), males (blue bars), and females (red bars). Panels represent: (**A**) TG/HDL-C, (**B**) TC/HDL-C, (**C**) LDL-C/HDL-C, (**D**) RC/HDL-C, (**E**) LCI. Asterisks indicate statistical significance between N and H groups within each sex category (**** *p* < 0.0001).

Figure 3 presents the median UAPR across normal and abnormal lipid ratio groups, stratified by sex. In all six panels, individuals with elevated lipid ratios consistently exhibited higher UAPR levels than those with normal ratios. Specifically, UAPR increased significantly in those with elevated TG/HDL-C (Figure 3A), TC/HDL-C (Figure 3B), LDL-C/HDL-C (Figure 3C), RC/HDL-C (Figure 3D) and Lipoprotein Combine Index (LCI) (Figure 3E). This pattern remained evident in both males and females, with females showing more pronounced UAPR elevations in the presence of abnormal ratios.

### 3.5. Significant Associations Between UAPR and Both Conventional Lipid Markers and Ratios

In Table 4, Spearman correlation analysis revealed significant associations between UAPR and both conventional lipid parameters and calculated lipid ratios. Among the individual lipid markers, the strongest positive correlations were observed with TG (r = 0.226, *p* < 0.0001), RC (r = 0.182, *p* < 0.0001), and non-HDL-C (r = 0.180, *p* < 0.0001). UAPR showed a moderate positive correlation found with LDL-C (r = 0.131, *p* < 0.0001) and TC (r = 0.074, *p* < 0.0001), while HDL-C demonstrated a significant inverse relationship (r = −0.314, *p* < 0.0001), indicating that lower HDL-C levels correspond to higher UAPR.

Stronger correlations were evident between UAPR and lipid ratios. The TG/HDL-C ratio (r = 0.298, *p* < 0.0001), TC/HDL-C ratio (r = 0.302, *p* < 0.0001) and LDL-C/HDL-C ratio (r = 0.284, *p* < 0.0001) all showed strong positive associations. RC/HDL-C ratio (r = 0.242, *p* < 0.0001) and LCI (r = 0.260, *p* < 0.0001) also demonstrated significant positive correlations with UAPR.

### 3.6. Univariate and Multivariable Regression Analyses of UAPR with Lipid Parameters

In univariate regression analyses (Table 5), UAPR showed significant positive associations with TG (β = 0.152, *p* < 0.0001) and RC (β = 0.166, *p* < 0.0001), while demonstrating a strong inverse relationship with HDL-C (β = −0.131, *p* < 0.0001). Modest associations were also observed with TC (β = 0.027, *p* = 0.018), LDL-C (β = 0.013, *p* = 0.023), and non-HDL-C (β = 0.030, *p* < 0.0001). After adjustment for age, sex, glycemic parameters (FBG, HbA1c), and renal function markers (creatinine, urea), the associations of UAPR with TG (β = 0.145, *p* < 0.001) and HDL-C (β = −0.126, *p* < 0.001) remained robust and statistically significant. In contrast, the associations with TC, LDL-C, non-HDL-C, and RC were attenuated and lost statistical significance, suggesting confounding effects of glycemic and renal factors.

### 3.7. Higher UAPR Values Are Linked to Greater Odds of Dyslipidemia and Atherogenic Lipid Ratios

**Figure 4:**
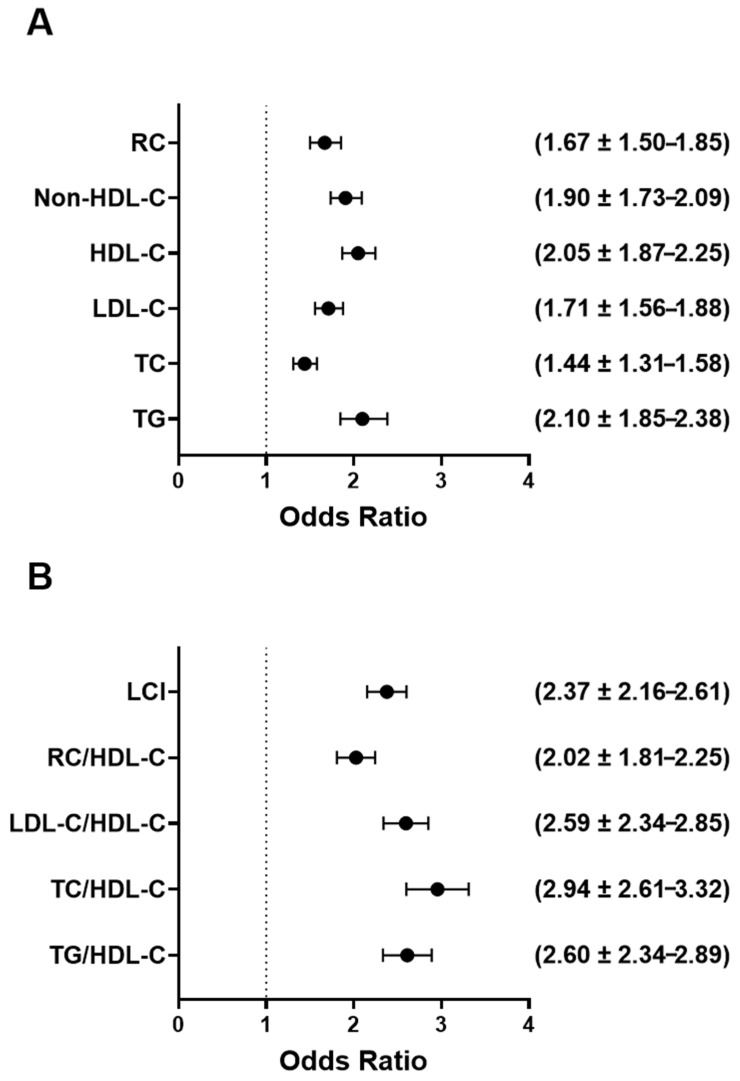
Forest plot of adjusted odds ratios for the association between elevated UAPR and risk of dyslipidemia: the odds ratios (OR) with 95% confidence intervals (CI) illustrate the strength of association between elevated UAPR and various forms of dyslipidemia. (**A**) The OR between elevated UAPR and abnormalities in standard lipid markers. (**B**) The OR between elevated UAPR and abnormalities in lipid-related ratios.

Figure 4 displays the odds ratios (ORs) and 95% confidence intervals for abnormal lipid parameters (Figure 4A) and elevated lipid ratios (Figure 4B) in relation to individuals in the high UAPR values. As shown in Figure 4A, Individuals with high UAPR showed increased odds of having elevated TG (OR = 2.10), TC (OR = 1.44), LDL-C (OR = 1.71), and non-HDL-C cholesterol (OR = 1.90). A notable association was also observed with reduced HDL-C levels, reflected by an odds ratio of 2.05, and with RC, which had an odds ratio of 1.67. Figure 4B demonstrates even stronger associations between high UAPR and abnormal lipid ratios. High UAPR was substantially associated with increased odds of having high TG/HDL-C, TC/HDL-C, LDL-C/HDL-C ratios (OR = 2.60, OR = 2.94 and OR= 2.59, respectively). High RC/HDL-C ratio was strongly associated with elevated UAPR (OR = 2.02). Lastly, the odds of having increased LCI was significantly elevated in individuals with high UAPR (OR = 2.37).

### 3.8. Subjects with High UAPR Exhibited a Higher Prevalence of Lipid Abnormalities

Table 6 presents the distribution of lipid parameters and atherogenic ratios among individuals with normal UAPR (N-UAPR) and high UAPR (H-UAPR). A consistent pattern was observed, where a greater proportion of individuals with lipid abnormalities were found in the H-UAPR group across nearly all markers. Notably, among participants with normal UAPR, the majority (89.31%) had TG levels within the normal range, while only 10.69% exhibited elevated TG levels. Among the high UAPR group; however, a notably smaller proportion (79.93%) had normal TG levels, and a higher proportion (20.07%) had elevated TG levels, indicating a relative shift toward hypertriglyceridemia in individuals with high UAPR. This pattern extended to other lipid markers. For TC, 67.98% of individuals with N-UAPR had normal TC, compared to 59.62% among those with H-UAPR. Elevated TC was more frequently observed in the H-UAPR group (40.38%) than in the N-UAPR group (32.02%). Similarly, elevated LDL-C was found in 44.93% of H-UAPR individuals versus 32.31% in the N-UAPR group. Regarding HDL-C, low levels were more prevalent in the H-UAPR group (52.4%) than in N-UAPR (35.0%). The percentage of elevated RC subjects was also greater in the H-UAPR group (28.76%) than in the N-UAPR group (19.49%).

Analysis of lipid ratios revealed further differences between individuals with N-UAPR and H-UAPR. For the TG/HDL-C ratio, a higher proportion of individuals with normal UAPR had elevated TG/HDL-C at 83.31%, compared to 65.74% among those with high UAPR. In contrast, a low TG/HDL-C ratio was more frequently observed among those with H-UAPR (34.26%) than those with normal UAPR (16.69%). Among individuals with normal UAPR, 88.83% had a high TC/HDL-C ratio, while only 73.01% of the high UAPR group fell into this category. Conversely, a greater proportion of individuals with high UAPR (26.99%) had TC/HDL-C < 5 compared to those with normal UAPR (11.17%). For the LDL-C/HDL-C, 77.57% of participants with normal UAPR had elevated values, compared to 57.20% in the high UAPR group. Similarly, those with high UAPR showed a higher prevalence of favorable LDL-C/HDL-C values at 42.80%, versus 22.43% in the normal UAPR group. For the RC/HDL-C, 82.82% of individuals with normal UAPR had values ≥ 0.56, while this ratio was elevated in 70.50% of the high UAPR group. A lower RC/HDL-C ratio was more prevalent in H-UAPR (29.50%) compared to N-UAPR (17.18%). Lastly, High LCI was present in 71.83% of individuals with normal UAPR and in only 51.82% of those with high UAPR. Meanwhile, a low LCI was more frequently seen among the high UAPR group (48.18%) than in the normal UAPR group (28.17%).

### 3.9. Discriminatory Performance of UAPR in Detecting Abnormal Lipid Parameters and Ratios

**Figure 5:**
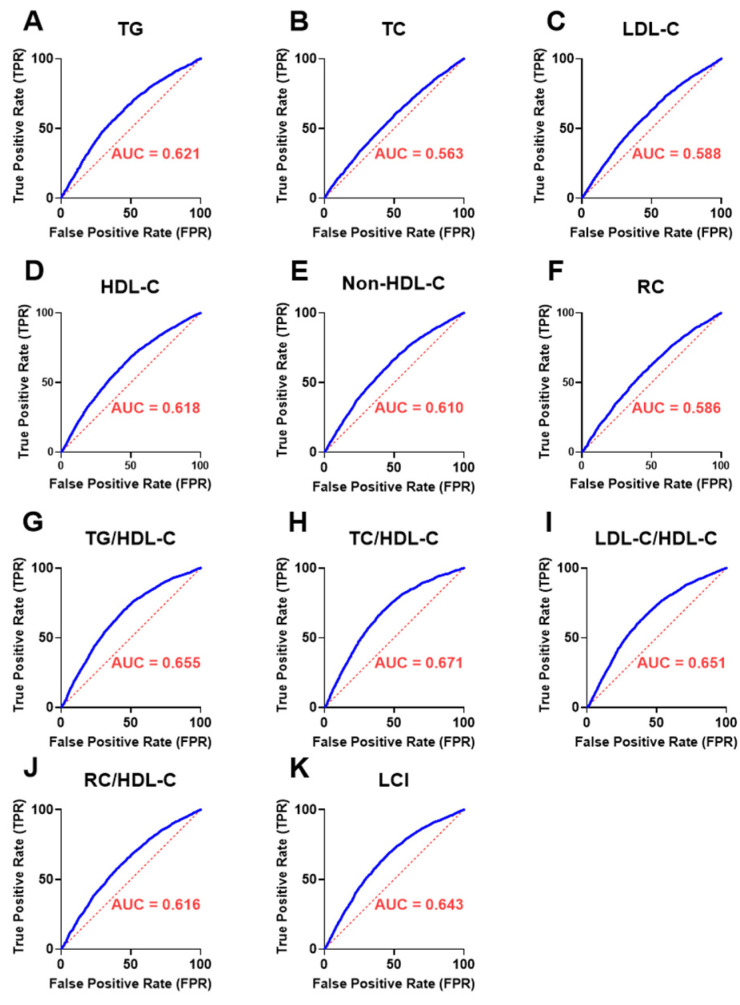
Analysis of the diagnostic accuracy of UAPR for lipid markers in both genders. ROC curve was conducted to assess the diagnostics capacity of UAPR in discriminating abnormalities across a range of lipid markers in all study cohort and among males and females separately as indicated. The ROC plots illustrate the performance of UAPR in relation to: (**A**) TG, (**B**) TC, (**C**) LDL-C, (**D**) HDL-C, (**E**) Non-HDL-C, (**F**) RC, (**G**) TG/HDL-C, (**H**) TC/HDL-C, (**I**) LDL-C/HDL-C, (**J**) RC/HDL-C, (**K**) LCI. The area under the curves (AUCs) and associated *P* values for each lipid parameter are summarized in the corresponding table.

Figure 5 presents receiver operating characteristic (ROC) curves evaluating the capacity of the UAPR in discriminating individuals with abnormal lipid parameters and elevated lipid ratios. UAPR showed statistically significant predictive value for all lipid abnormalities, with area under the curve (AUC) spanning from 0.563 to 0.681 (*p* < 0.001 for all). Among the individual lipid parameters, the strongest discrimination was observed for high TG (Figure 5A, AUC = 0.621) and low HDL-C (Figure 5D, AUC = 0.618), while moderate AUCs were noted for TC, LDL-C, non-HDL-C, and RC. Furthermore, UAPR demonstrated even stronger predictive power for lipid-derived ratios, with the highest AUCs seen for TG/HDL-C (Figure 5G, 0.655), LDL-C/HDL-C (Figure 5I, 0.671), and TC/HDL-C (Figure 5H, 0.651). The LCI and RC/HDL-C also exhibited moderate discriminatory performance.

## 4. Discussion

This study investigated the linkage between the uric acid to platelet ratio (UAPR) and various lipid parameters and lipid-related ratios in a broad population-based sample. Our findings consistently demonstrate that individuals with elevated UAPR values exhibit a significantly higher prevalence of lipid abnormalities, particularly reduced high-density lipoprotein cholesterol (HDL-C) and elevated concentrations of low-density lipoprotein cholesterol (LDL-C), non-HDL cholesterol, total cholesterol (TC), triglycerides (TG), and remnant cholesterol (RC). Additionally, UAPR was strongly associated with a range of atherogenic lipid ratios, including TG/HDL-C, TC/HDL-C, LDL/HDL-C, RC/HDL-C, and the Lipoprotein Combine Index (LCI). Correlation analysis further supported these associations, revealing stronger links between UAPR and lipid ratios compared to individual lipid markers. Subjects in the highest UAPR tertile had significantly increased odds of exhibiting both conventional and ratio-based lipid abnormalities. ROC curve analyses demonstrated moderate yet significant discriminative ability of UAPR for detecting dyslipidemia, particularly for low HDL-C, high TG, TG/HDL and LCI. Collectively, the results suggest that UAPR serves as a practical and economic indicator of atherogenic lipid burden underscoring its potential role in cardiovascular risk stratification.

The present analysis from a Saudi cohort highlights the variable prevalence of dyslipidemia across individual lipid components and underscores significant sex-specific differences. Among the studied population, HDL-C and LDL-C appeared as the most prevalent lipid abnormalities, affecting approximately 42.2% and 38% of participants, respectively. This aligns with global and regional data identifying LDL-C and HDL-C as the most common dyslipidemic phenotypes and key contributors to atherogenic risk profiles [47,48]. Interestingly, the prevalence of low HDL-C, high LDL-C, total cholesterol (TC), and non-HDL-C levels was notably greater in females than in males, suggesting a potential sex-linked predisposition or differing exposure to lifestyle and hormonal factors influencing lipid metabolism in this population [49,50]. A large-scale study conducted between 2007 and 2018 found that women exhibited poorer lipid control compared to men, primarily due to lower adherence to statin therapy, which was attributed to a higher incidence of perceived or actual side effects [51]. These outcomes point to the necessity of adopting sex-specific approaches in lipid management and cardiovascular risk assessment.

Prior literature supports the connection between uric acid and lipid abnormalities. Dyslipidemia is positively associated with hyperuricemia in adult population of Bangladesh, Italy, Korea, China and USA [52,53,54,55,56]. Moreover, evidence from large-scale Chinese cohort shows that hyperuricemia is positively linked with high TG, high LDL-C and low HDL-C [54]. A study by Alaql et al. in Saudi Arabia evaluated the link between hyperuricemia and lipid parameters in adult sample, revealing a significant association with TG but not with total cholesterol [57]. Although our study did not delve into the mechanistic pathways linking hyperuricemia to hyperlipidemia, accumulating evidence from animal ana human studies suggests that visceral adipose tissue might function as a critical intermediary. Human adipose tissue has been shown to secrete hypoxanthine, a purine precursor of uric acid, particularly under hypoxic conditions [58]. Moreover, insulin resistant adipose tissue has been independently linked to a higher risk of developing hyperuricemia [59]. Experimental studies in obese mouse models demonstrated that enhanced xanthine oxidoreductase (XOR) activity in adipose tissue, which accelerates uric acid production through hypoxia-driven purine catabolism [60]. These findings underscore the pathophysiological role of dysfunctional insulin resistant and hypoxic adipose tissue in promoting uric acid overproduction, thereby linking obesity to hyperuricemia to hyperuricemia and, potentially, to lipid abnormalities.

Our results demonstrate that UAPR is strongly linked to elevated TG and diminished HDL-C independent of age, sex, glycemia, and renal function, suggesting that this index reflects an atherosclerotic phenotype of dyslipidemia. Indeed, elevated TG represents a hallmark of atherogenic dyslipidemia, frequently associated with insulin resistance, visceral adiposity, and metabolic syndrome, while diminished HDL-C is a central lipid abnormality that contributes substantially to cardiovascular risk [61]. The persistence of these associations even after adjusting for glycemic and renal parameters emphasizes the robustness of UAPR as an independent marker of lipid abnormalities. Notably, our findings indicate that low HDL-C showed the most pronounced correlation with UAPR, surpassing associations observed with other conventional lipid measures. This suggests that UAPR may be particularly sensitive to changes in HDL-C levels, which are critically involved in reverse cholesterol transport and possess anti-inflammatory and antioxidant properties. This observation may reflect the influence of elevated uric acid, diminished platelets or both. While uric acid is recognized for its antioxidant properties and its role on counteracting against oxidative stress, growing evidence suggests that its role is concentration-dependent transitioning from antioxidant to pro-oxidant effects at elevated levels [62,63]. On the other hand, platelet count inversely contributes to the UAPR index. Platelets are increasingly recognized not only for their role in thrombosis but also in inflammation and lipid transport. Low platelet counts, or platelet exhaustion in chronic metabolic states, may reflect systemic inflammatory burden or bone marrow suppression, both of which have been linked to dyslipidemia and metabolic syndrome [64,65]. Since UAPR integrates both uric acid and platelet count, it may serve as a composite indicator of redox imbalance, inflammatory burden, and metabolic dysfunction, all of which are linked to lower HDL-C levels. Although our study did not assess HDL-C functionality, which includes its antioxidant, anti-inflammatory, and cholesterol efflux capacities, this aspect warrants further investigation to better understand the clinical implications of the UAPR and HDL-C association.

Our data further revealed that UAPR had a stronger discriminatory performance for lipid ratios than for single lipid parameters. This finding is relevant because lipid indices such as LDL-C/HDL-C, TC/HDL-C, and TG/HDL-C are often considered more predictive of cardiovascular events than isolated lipid levels, although their added clinical value remains debated and not universally accepted [66,67,68]. Notably, UAPR performed particularly well in identifying individuals with high TG/HDL-C ratio. These ratios are sensitive indicators of remnant lipoproteins and subclinical insulin resistance. There appears to be a correlation between serum uric acid and insulin resistance in various populations. Data from large-scale cohort such as MASHAD study in Iran and the Finnish GOAL cohort, showed that hyperuricemia is independently associated with higher fasting glucose, insulin and surrogate indices of insulin resistance such as HOMA-IR and TyG even in non-diabetic populations [69,70]. The proposed mechanisms are multifactorial but suggest that insulin resistance might impair renal excretions of uric acid via enhancing tubular reabsorption through sodium-urate cotransporters and promotes endogenous uric acid production through increased purine metabolism, particularly under hyperinsulinemic conditions [71,72]. While insulin resistance appears to be the primary driver, elevated uric acid may also contribute to worsening IR by inducing endothelial dysfunction, oxidative stress, and impaired insulin signaling in adipose tissue [70,72]. Interestingly, lifestyle-related IR markers show a stronger association with hyperuricemia than dietary indices alone, suggesting that metabolic dysfunction, rather than diet per se, plays a more dominant role [69]. Collectively, these findings indicate a bidirectional relationship, where insulin resistance initiates and sustains hyperuricemia, while elevated uric acid exacerbates the metabolic disturbance, reinforcing the link between impaired glucose metabolism and altered purine handling in the development of cardiometabolic disorders.

From a clinical standpoint, our results suggest that UAPR may serve as a useful, integrative biomarker in routine assessments to flag individuals at elevated cardiometabolic risk. UAPR utilizes two widely available, inexpensive laboratory measures, making it particularly valuable in resource-limited settings. Given its association with both traditional lipid markers and advanced atherogenic ratios, UAPR could serve as a complementary tool alongside lipid panels to enhance early risk stratification and guide preventive interventions. However, it is important to note that while the AUC values observed in this study were statistically significant, they demonstrated only moderate discriminative ability (ranging from approximately 0.60 to 0.68). This likely reflects the multifactorial etiology of dyslipidemia and suggests that UAPR is best utilized as part of a broader, multi-marker risk assessment strategy rather than as a standalone diagnostic tool.

This study benefits from several notable strengths, including its very large-scale cohort, low analytical variability due to automated data acquisition and analysis, and the inclusion of a detailed lipid profile. Nevertheless, several limitations must be acknowledged. First, the cross-sectional nature of the study precludes causal inferences. While associations are strong and biologically plausible, longitudinal studies are needed to confirm whether elevated UAPR precedes and predicts the development of dyslipidemia or cardiovascular events. Second, the inability to adjust for potential confounding variables, such as BMI and medication history, represents a key limitation. This was primarily due to the retrospective design of the study and the absence of several critical clinical variables in the dataset. Future studies should confirm these findings prospectively and evaluate the clinical value of UAPR in risk prediction models.

## 5. Conclusions

In summary, this study demonstrates that higher UAPR is independently associated with elevated triglycerides and reduced HDL-C, underscoring its utility as a marker of atherogenic dyslipidemia. Owing to its simplicity and accessibility, UAPR may serve as a practical supplementary tool for cardiovascular risk screening, particularly in resource-limited settings. Future prospective and interventional studies are warranted to validate its predictive value and define its role in cardiometabolic risk assessment and management.

## Figures and Tables

**Table 1:** Baseline biochemical and hematological parameters of the study cohort.

Variable	Median (±IQR)
Age (Years)	37 (32–46)
Sex (female), %	60.25
Hemoglobin (g/dL)	14.2 (13–15.6)
Red Cell Count (×10^12^/μL)	5.24 (4.86–5.67)
Total Leukocytic Count (×10^9^/μL)	5.65 (4.57–6.97)
Fasting Plasma Glucose (mg/dL)	92 (86–99)
Glycated hemoglobin A1c (HbA1c) %	5.3 (5.1–5.3)
Creatinine (mg/dL)	0.70 (0.60–0.85)
Urea (mg/dL)	22 (16–27)
Triglycerides (mg/dL)	93 (70–127)
Total Cholesterol (mg/dL)	187 (165–209)
Low-Density Lipoprotein Cholesterol (mg/dL)	120 (99–141)
High-Density Lipoprotein Cholesterol (mg/dL)	47 (41–55)
Non-HDL Cholesterol (mg/dL)	137 (115–161)
Remnant Cholesterol (mg/dL)	16 (10–23)
Alanine Aminotransferase (U/L)	18 (13–27)
Aspartate Aminotransferase (U/L)	18 (15–23)
Albumin in Serum (g/dL)	4.2 (4–4.5)
Erythrocyte Sedimentation Rate (mm/h)	8 (5–15)
25-Hydroxyvitamin D (ng/mL)	14.3 (9.9–21.9)

**Table 2:** Sex-specific distribution of dyslipidemia in the study cohort.

Lipid Abnormality	Overall Prevalence (%)	Male Prevalence (%)	Female Prevalence (%)	*p* Value
High TG	14.59	15.71	13.84	0.0243
High TC	35.5	35.92	35.22	0.5423
High LDL-C	37.55	39.73	36.11	0.0014
Low HDL-C	42.22	23.63	54.5	<0.0001
High Non-HDL-C	36.7	38.96	35.22	0.0009
High RC	23.34	23.5	23.23	0.7999
High TG/HDL-C Ratio	23.99	25.93	22.72	0.0013
High TC/HDL-C Ratio	17.75	20.21	16.13	<0.0001
High LDL-C/HDL-C Ratio	30.9	34.46	28.54	<0.0001
High RC/HDL-C Ratio	22.3	23.08	21.78	0.1849
High LCI	36.49	39.8	34.3	<0.0001

Abbreviations: TG, triglycerides; TC, total cholesterol; LDL-C, low-density lipoprotein cholesterol; HDL-C, high-density lipoprotein cholesterol; Non-HDL-C, non–high-density lipoprotein cholesterol; RC, remnant cholesterol; TC/HDL-C, total cholesterol to HDL-C ratio; LDL/HDL-C, LDL-C to HDL-C ratio; TG/HDL-C, triglycerides to HDL-C ratio; RC/HDL-C, remnant cholesterol to HDL-C ratio; LCI, lipoprotein combine index.

**Table 3:** Comparison of lipid panels and their derived ratios across UAPR tertiles.

Lipid Parameters and Ratios	T1	T2	T3	*p* Value
TG	82 (63–110)	94.5 (71–127)	106 (78–142.3)	<0.0001
TC	204 (164–183)	208 (165–187.5)	214.3 (165–191)	<0.0001
LDL-C	97 (114–133)	100 (120.5–141)	102 (126–147)	<0.0001
HDL-C	45 (52–60)	41 (47–55)	37 (43–51)	<0.0001
Non-HDL-C	129 (111–150)	138 (117–160)	146 (120–170)	<0.0001
RC	14 (9–20)	16 (10–23)	18 (12–25)	<0.0001
TG/HDL-C	1.60 (1.11–2.27)	2.00 (1.38–2.91)	2.42 (1.66–3.56)	<0.0001
LDL-C/HDL-C	2.19 (1.73–2.75)	2.53 (1.98–3.13)	2.86 (2.23–3.54)	<0.0001
TC-C/HDL-C	3.49 (2.96–4.14)	3.90 (3.27–4.63)	4.31 (3.58–5.13)	<0.0001
RC/HDL-C	0.26 (0.16–0.43)	0.33 (0.2–0.53)	0.41 (0.25–0.64)	<0.0001
LCI	9.65 (5.76–15.9)	13.01 (7.55–21.91)	16.96 (9.30–28.57)	<0.0001

Abbreviations: TG, triglycerides; TC, total cholesterol; LDL-C, low-density lipoprotein cholesterol; HDL-C, high-density lipoprotein cholesterol; Non-HDL-C, non–high-density lipoprotein cholesterol; RC, remnant cholesterol; TC/HDL-C, total cholesterol to HDL-C ratio; LDL/HDL-C, LDL-C to HDL-C ratio; TG/HDL-C, triglycerides to HDL-C ratio; RC/HDL-C, remnant cholesterol to HDL-C ratio; LCI, lipoprotein combine index.

**Table 4:** Correlation analysis between UAPR and lipid profile components and derived ratios.

Lipid Marker	r (q-Values ^a^)	Lipid Ratio	r (q-Values)
TG	0.226 (<0.0001)	TG/HDL-C	0.298 (<0.0001)
TC	0.074 (<0.0001)	TC/HDL-C	0.302 (<0.0001)
LDL-C	0.131 (<0.0001)	LDL-C/HDL-C	0.284 (<0.0001)
HDL-C	−0.314 (<0.0001)	RC/HDL-C	0.242 (<0.0001)
Non-HDL-C	0.180 (<0.0001)	LCI	0.260 (<0.0001)
RC	0.182 (<0.0001)		

^a^ q-values were the *p* values corrected for multiple comparisons using the Benjamini-Hochberg false-discovery rate. Abbreviations: TG, triglycerides; TC, total cholesterol; LDL-C, low-density lipoprotein cholesterol; HDL-C, high-density lipoprotein cholesterol; Non-HDL-C, non–high-density lipoprotein cholesterol; RC, remnant cholesterol; TC/HDL-C, total cholesterol to HDL-C ratio; LDL-C/HDL-C, LDL-C to HDL-C ratio; TG/HDL-C, triglycerides to HDL-C ratio; RC/HDL-C, remnant cholesterol to HDL-C ratio; LCI, lipoprotein combine index.

**Table 5:** Univariate and multivariable regression analyses of UAPR with lipid parameters.

Lipid Parameter	Univariate β (95% CI)	*p* Value	Multivariable β (95% CI)	*p* Value
TG	0.15 (0.12–0.18)	<0.0001	0.14 (0.11–0.18)	<0.0001
TC	−0.01 (−0.02–0.01)	0.0004	−0.01 (−0.03–0.01)	0.42
LDL-C	0.01 (0.00–0.02)	0.0227	0.01 (−0.02–0.03)	0.589
HDL-C	−0.13 (−0.16–0.10)	<0.0001	−0.12 (−0.15–0.08)	<0.0001
non-HDL-C	0.03 (0.02–0.04)	<0.0001	0.01 (−0.01–0.04)	0.381
RC	0.17 (0.14–0.20)	<0.0001	−0.01 (−0.47–0.44)	0.9613

Multivariable models were adjusted for age, sex, fasting blood glucose, glycated hemoglobin (HbA1c), serum creatinine, and urea. Abbreviations: TG, triglycerides; TC, total cholesterol; LDL-C, low-density lipoprotein cholesterol; HDL-C, high-density lipoprotein cholesterol; Non-HDL-C, non–high-density lipoprotein cholesterol; RC, remnant cholesterol.

**Table 6:** Stratified distribution of UAPR levels across lipid profile categories.

Lipid Parameters and Ratios		N-UAPR (%)	H-UAPR (%)
Triglycerides (TG)	<150	89.31	79.93
	≥150	10.69	20.07
TG/HDL-C Ratio	<3	83.31	65.74
	≥3	16.69	34.26
Total Cholesterol (TC)	<200	67.98	59.62
	≥200	32.02	40.38
TC/HDL-C Ratio	<5	88.83	73.01
	≥5	11.17	26.99
LDL-C	<130	67.69	55.07
	≥130	32.31	44.93
LDL-C/HDL-C Ratio	<3	77.57	57.2
	≥3	22.43	42.8
HDL-C	≥40 in males, ≥50 in females	65.03	47.59
	<40 in males, <50 in females	34.97	52.41
Non-HDL-C	<150	69.54	54.51
	≥150	30.46	45.49
Remnant Cholesterol (RC)	≤23	80.51	71.24
	>23	19.49	28.76
RC/HDL-C Ratio	<0.56	82.82	70.5
	≥0.56	17.18	29.5
Lipoprotein Combine Index (LCI)	<16	71.83	51.82
	≥16	28.17	48.18

Abbreviations: LDL-C, low-density lipoprotein cholesterol; HDL-C, high-density lipoprotein cholesterol; Non-HDL-C, non–high-density lipoprotein cholesterol; TC/HDL-C, total cholesterol to HDL-C ratio; LDL/HDL-C, LDL-C to HDL-C ratio; TG/HDL-C, triglycerides to HDL-C ratio;; RC/HDL-C, remnant cholesterol to HDL-C ratio.

## Data Availability

The datasets generated and analyzed during the current study are available from the corresponding author, Y.A., upon reasonable request and subject to approval by Elta Medical Laboratories.
